# *Ceratophysella* species from mushrooms in China (Collembola, Hypogastruridae)

**DOI:** 10.3897/zookeys.822.30880

**Published:** 2019-02-05

**Authors:** Wanda Maria Weiner, Zhijing Xie, Yu Li, Xin Sun

**Affiliations:** 1 Institute of Systematics and Evolution of Animals, Polish Academy of Sciences, Sławkowska 17, 31-016 Kraków, Poland Institute of Systematics and Evolution of Animals, Polish Academy of Sciences Kraków Poland; 2 Key laboratory of Wetland Ecology and Environment, Northeast Institute of Geography and Agroecology, Chinese Academy of Sciences, Changchun 130102, China Northeast Institute of Geography and Agroecology, Chinese Academy of Sciences Changchun China; 3 University of Chinese Academy of Sciences, Beijing 100049, China University of Chinese Academy of Sciences Beijing China; 4 Engineering Research Center of Chinese Ministry of Education for Edible and Medicinal Fungi, Jilin Agricultural University, Changchun 130118, China Jilin Agricultural University Changchun China; 5 J.F. Blumenbach Institute of Zoology and Anthropology, University of Göttingen, 37073 Göttingen, Germany University of Göttingen Göttingen Germany

**Keywords:** *Ceratophysellaskarzynskii* sp. n., key, new species, taxonomy

## Abstract

Four species of the genus *Ceratophysella* living on mushrooms are reported from China, including a new species, *Ceratophysellaskarzynskii* Weiner & Sun, **sp. n.**, which is described from alpine mushrooms. The new species belongs to the *Ceratophysella* group of species with a dorsal chaetotaxy of type B and differs from the other species in a combination of characters. *Ceratophysellaskarzynskii***sp. n.** is distinguished by its small body size (maximum length 1.09 mm), number of peg-like s-chaetae (30–32) in the ventral sensory file, the trilobed apical vesicle of antennal segment IV, five modified chaetae on dens, and serrated dorsal chaetae. A key to the Chinese species of the genus has been provided.

## Introduction

The genus *Ceratophysella* Börner, 1932 is distributed worldwide, having more than 130 species (Bellinger et al. 1996–2018). The main diagnostic characters for the genus are the pigmented body, 8 + 8 ocelli, body chaetae mostly differentiated into micro- and macrochaetae, an eversible integumental sac usually present between antennal segments III and IV, the ventral side of antennal segment IV with a sensory file often well-developed of short, erect, curved, and flattened at tips s-chaetae, unguiculus with broad basal lamella, furca well developed, mucro usually boat-like with a spoon-like apex and distinct lamella, and anal spines usually long and curved.

Until now, fourteen species of the genus *Ceratophysella* have been reported from China (Zhao 1992, Shen 1993, Liu et al. 1998, Zhao et al. 1997, Jia et al. 2010). As a common group of Collembola living on mushrooms, species have often caused significant economic damage in China (Wei 2002; Zhu 2012). Within a large collection of the mushroom Collembola in China, three known species, *C.communis* (Folsom, 1898), *C.denticulata* (Bagnall, 1941), *C.liguladorsi* (Lee, 1974), and the new species described here, *C.skarzynskii* sp. n., are reported in the present paper.

## Materials and methods

Specimens were collected by hand using a brush and stored in ethanol; they were then cleared in lactic acid and KOH, and mounted in Marc André II medium. Drawings and measurements were made using a phase contrast microscope LEICA DM2500 equipped with a camera lucida.

Mushroom species were determined by the third author, Yu Li.

**Abbreviations used in the descriptions**:

**Abd.** abdominal segments,

**Ant.** antennal segments,

**av** apical vesicle

**AIIIO** sensory organ of Ant. III,

**l.p.** lateral processus on labial palp,

**ms** s-microsetae (ms) (microsensillum),

**or** subapical organite

**PAO** postantennal organ,

**S** sensillum,

**s-chaetae** sensorial chaetae on Th. and Abd.

**Th.** thoracic segments,

**VT** ventral tube,

**IGA-CAS**Northeast Institute of Geography and Agroecology, Chinese Academy of Sciences;

**ISEA-PAS** Institute of Systematics and Evolution of Animals, Polish Academy of Sciences.

Terminology for the descriptions follows that given in Fjellberg (1984, 1999), Babenko et al. (1994), and Thibaud et al. (2004).

## Taxonomy

### Ceratophysella skarzynskii

Taxon classificationAnimaliaCollembolaHypogastruridae

Weiner & Sun sp. n.

http://zoobank.org/BA3F1CD5-D62C-4AFA-8450-26FB928994CA

**Figure 1.**
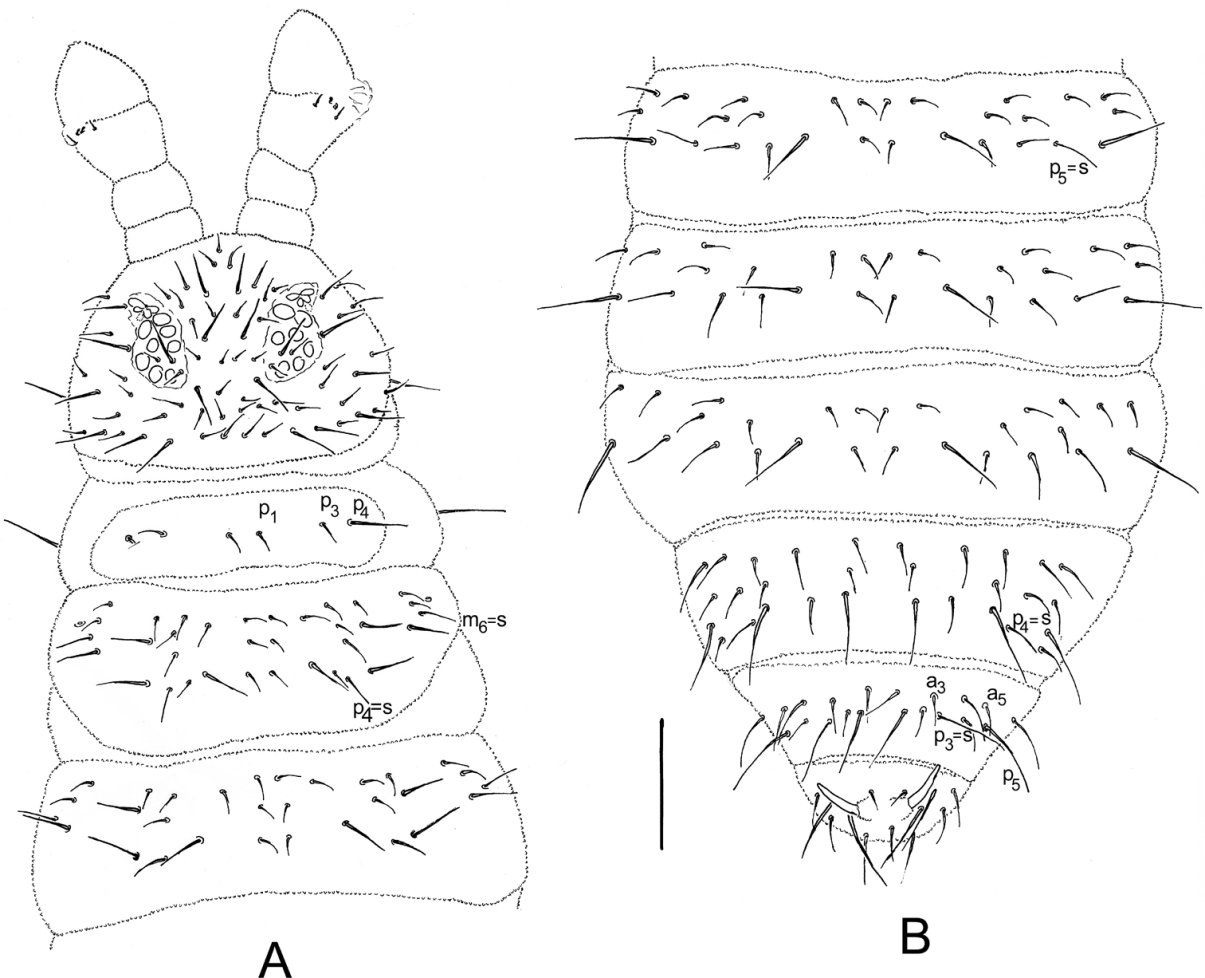
*Ceratophysellaskarzynskii* sp. n. **A** Chaetotaxy of head and Th. I–III **B** Chaetotaxy of Abd. I–VI. Scale bars: 0.1 mm.

**Figure 2.**
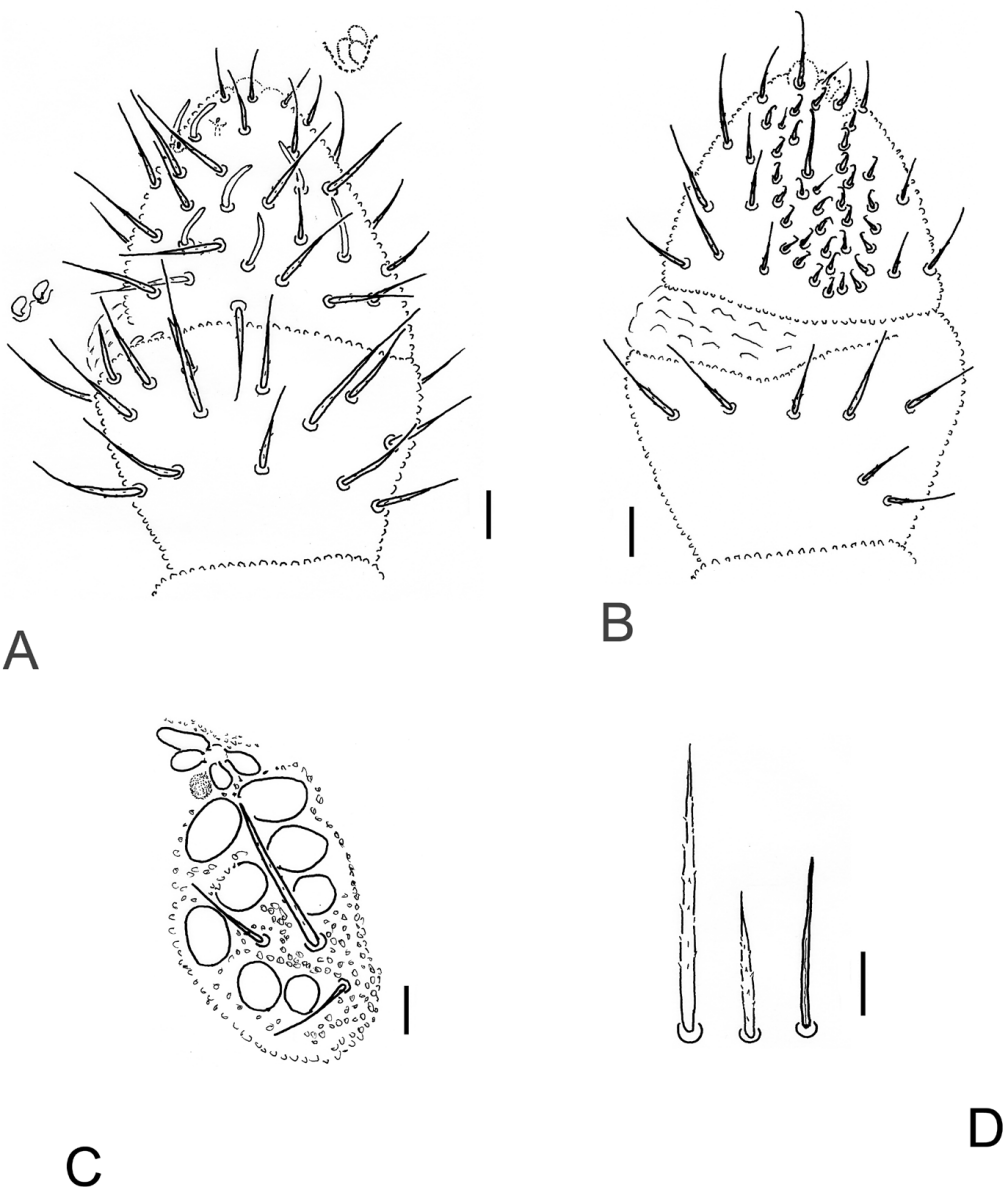
*Ceratophysellaskarzynskii* sp. n. **A** ant. III and IV dorsal **B** ant. III and IV dorsal **C**PAO and eyes **D** macro-, microchaetae and s-chaeta. Scale bars: 0.01 mm.

**Figure 3.**
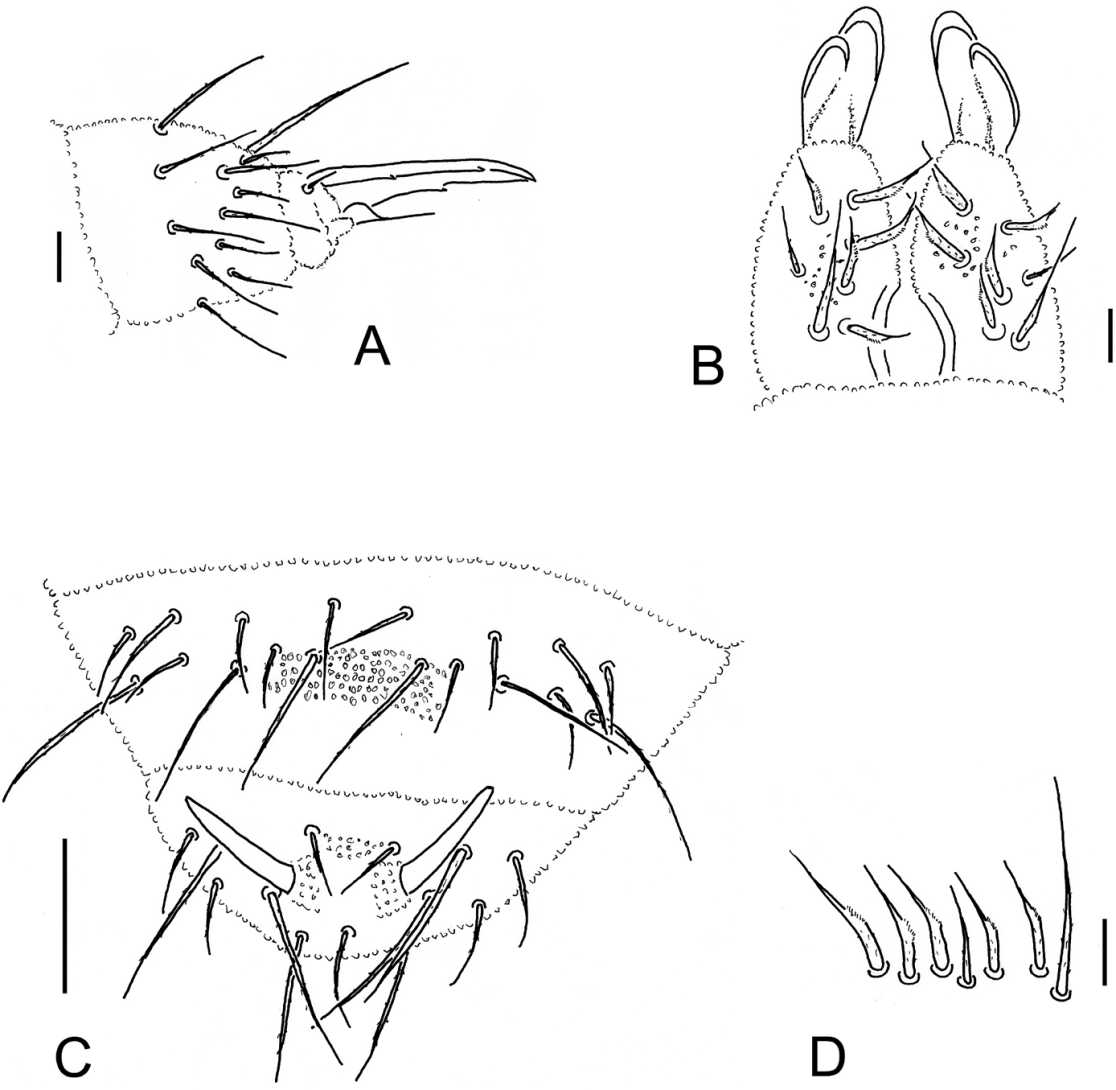
*Ceratophysellaskarzynskii* sp. n. **A** tibiotarsus and claw III **B** dens and mucro **C** abd. V and VI **D** set of dens chaetae. Scale bars: 0.01 mm.

Figs 1 , 2 , 3 , Table 1

#### Type material.

***Holotype*** : preadult male, China: Jilin: Changbai Mountains, alt. 2000 m, on *Russula* sp., leg. Xin Sun, 29 July 2015. ***Paratypes***: 10 females and one juvenile, the same data as holotype. Type material: the holotype and 8 paratypes are housed in IGA-CAS, China, two paratypes in ISEA-PAS, Poland.

#### Diagnosis.

Dorsal chaetotaxy of type B with serrated chaetae. Maximal length 1.09 mm. Antennal segment IV with bi- or trilobed apical vesicle and ventral sensory file with 30–32 peg-like s-chaetae. Dens with seven chaetae, five of them modified.

#### Description.

Body length 0.9–1.09 mm (holotype: 1.07 mm). Body colour violet or blue in alive specimens, grey or grey-black in alcohol, ventrally pale. Granulation rather coarse, 10–14 granules between chaetae p_1_ on Abd. V (Yosii’s parameter).

*Antennae*. Ant. IV with bilobed or trilobed apical vesicle (av), subapical organite (or), dorso-lateral microsensillum (ms), seven cylindrical, subequal sensilla (dorsal S0, S1–4, dorsolateral S7–8), ca. 30 small, peg-like sensilla and one subcylindrical sensillum in ventral sensory file (sensory rasp) (Fig. 2A, B). Ant. III-organ with two long (external) and two short (internal) curved sensilla (Fig. 2A). Microsensillum on ant. III present. Eversible sac between Ant. III–IV present (Fig. 2B). Ant. I with seven chaetae, Ant. II with 13 chaetae.

*Head*. Ocelli 8 + 8. Postantennal organ 1.5 times as large as single ocellus with four lobes of which the anterior pair is larger than the posterior pair (Fig. 2C). Accessory boss present (Fig. 2C).

*Labrum* with 5, 5, 4 chaetae, four prelabrals present. Head of maxilla of the *C.armata*type. Maxillary outer lobe with two sublobal hairs. Labium of the *C.armata* type, with five papillae (A–E) and six proximal chaetae. Guard chaetae a_1_, b_1–2_, d_2_, e_2_ and lateral processus (l.p.) as accessory papillae with short terminal sensillum. Guards b_3–4_, d_3–4_, and e_1–6_ with long sensilla. Dorsal guards b_3–4_, d_3–4_, and e_3_ distally expanded and flattened.

*Chaetotaxy*. Differentiation of dorsal chaetae into micro-/meso- and macrochaetae quite distinct (Figs 1A, 1B, 2D, 3C). Arrangement of chaetae on head typical for the genus, spine-like chaetae absent. Cephalic chaetae d_2, 4_, v_2_, p_3,4_, g_1, 5_, l_01_, l_11_ as macrochaetae. Dorsal chaetotaxy of B type (sensu Gisin 1947, Bourgeois and Cassagnau 1972, and Babenko et al. 1994) (Fig. 1B). Chaetae of medium length, pointed and serrated. Th. I with macrochaetae p_4_, without p_2_. Th. II–III with macrochaetae p_2_ (shifted forward), p_5, 6_, m_5_, chaetae m_4_ and m_5_ (Th. II with m_4’_and microsensillum ms), chaetae p_4_ m_6_ as sensorial chaetae s, chaetae a_2_ as long as a_3_. Abd. I–III with macrochaetae p_2,6_, sensorial chaetae s = p_5_. Abd. IV with macrochaetae p_1, 3, 6_, s-chaetae as p_4_. Abd. V with macrochaetae p_1, 5_, 4 + 4 a-chaetae inside two macrochaetae p5 (a_2, 2’_ absent, chaeta a_5_straight above p_5_) (Figs 1B, 3C). Body s-chaetae relatively long, but shorter than macrochaetae, only on Abd. V as long as macrochaetae p_1, 5_ (Figs 1A, B, 3C).

*Tibiotarsi* I, II, III with 19, 19, 18 chaetae respectively, including one acuminate tenent hair A_1_ each, femora with 13, 13, 12 chaetae, trochanters with 7, 7, 7 chaetae, coxae with 3, 7, 8 chaetae, subcoxae II with 0, 3, 3 chaetae, subcoxaeI with 1, 2, 3 chaetae. Claws with inner tooth and two pairs of lateral teeth. Empodial appendage with broad basal lamella and apical filament reaching slightly below inner tooth (ratio empodial filament: inner edge of claw = 0.47) (Fig. 3A). Ventral tube with 4 + 4 chaetae.

*Retinaculum* with 4 + 4 teeth. Furca well developed. Ratio dens + mucro: inner edge of claw III = 2.11: 1, ratio dens: mucro = 1.79: 1. Cuticular skeleton of furca well visible. Dens with uniform granulation and seven dorsal chaetae of which five are modified, two strongly thickened and three moderately so; basal macrochaeta longer than others chaetae, shorter than dens (3/5 of its length). Mucro boat-like with clear outer lamella (Fig. 3B, D).

*Anal spines* as long as inner edge of claw III slightly curved, situated on basal papillae, colourless (Figs 1B, 3C).

#### Etymology.

The species is cordially dedicated to our colleague and friend Dr Dariusz Skarżyński, a prominent Polish specialist in Hypogastruridae, Collembola.

#### Remarks.

The new species belongs to the *armata*-group of species, group B (Abd. tergum IV with p_1_ as macrochaeta) and subgroup B2 (Abd. tergum IV without chaeta p_3_) (Bourgeois and Cassagnau 1972). Among the species which could belong to this subgroup,*C.skarzynskii* is most similar to four species: *C.denisana* (Yosii, 1956), *C.empodialis* Babenko, 1994 (in Babenko et al. 1994), *C.longispina* (Tullberg, 1876), and *C.scotica* (Carpenter & Evans, 1899), due to the absence of transformed into spines, spine-like chaetae or spine-like integumentary protuberance on the head orAbd. V. They differ in the shape of the apical vesicle, the number of modified chaetae on the dens, the number of peg-like chaetae in the ventral sensory file on Ant. IV, length of empodial appendage,in the type of dorsal chaetae (serrated or smooth), and in the number of sublobal hairs on maxillary palp (one or two). A comparison of these species is presented in Table 1.

**Table 1.** Morphological characters for *C.skarzynski* sp. nov. and similar species: *C.denisana* Yosii, 1954 (Yosii 1956), *C.empodialis* Babenko, 1994, *C.longispina* (Tullberg, 1876) and *C.scotica* (Carpenter & Evans, 1899) after authors and Babenko et al. (1994).

Species/characters	* C. denisana *	* C. skarzynskii *	* C. empodialis *	* C. longispina *	* C. scotica *
Maximal body size (mm)	1.20	1.09	1.80	2.00	2.00
Yosii’ parameter	14–16 (20)	10–14	8–13	12–16	13–15
Ant.IV: apical vesicle	trilobed	trilobed	simple/slightly bilobed	simple/slightly bilobed	simple
Ant.IV: number of peg-like chaetae in ventral file	>50	30–32	20	max. 15	15–20
Maxillar palp: number of sublobal hairs	2	2	2	2	1
Dorsal chaetae	smooth	serrated	rather smooth	serrated	rather smooth
Th.II–III: length of chaetae s = p_4_/p_3_	p_4_ >p_3_	p_4_ >p_3_	p_4_ >p_3_	p_4_≈p_3_	p_4_≈p_3_
Abd. V: chaetae s = p_3_/p_1_	p_3_≈p_1_	p_3_≈p_1_	p_3_<p_1_	p_3_≈p_1_	p_3_<p_1_
Tibiotarsial tenent hair	?	pointed	pointed	?	pointed
Empodial appendage : inner edge of claw	±½	±½	±1¼	±½	±1
Empodial basal lamella : inner edge of claw	1/4	1/5	1/5	?	1/5
Lateral teeth of claw	basal 3 pairs	2 pairs	2 pairs	2 pairs	2 pairs
Chaetae on dens: total number/numer of modified chaetae	7/5	7/5	7/2	7(8)/2	7/2

### Ceratophysella communis

Taxon classificationAnimaliaCollembolaHypogastruridae

(Folsom, 1898)

Acorutes communis Folsom, 1898: 52.
Syn: Hypogastrurayuasai Yosii, 1954: Yosii 1960.

#### Studied material.

China: Henan: Zhengzhou, on *Pleurotusostreatus*, 7 specimens on slides and 20 in alcohol, leg. Xin Sun, 05 Dec 2014; China: Henan: Zhumadian, on *Pleurotusostreatus*, 9 specimens on slides and 50 in alcohol, leg. ZhijingXie, 10 Nov 2017; China: Zhejiang: Pinghu, on *Pleurotusostreatus*, 13 specimens on slides and 100 in alcohol, leg. Xin Sun, 07 Dec 2014; China: Sichuan: Qingchuan, on *Morchellaesculenta*, 4 specimens on slides and 30 in alcohol, leg. Zhijing Xie, 19 Mar 2017.

### Ceratophysella denticulata

Taxon classificationAnimaliaCollembolaHypogastruridae

(Bagnall, 1941)

Achorutes denticulatus Bagnall, 1941: 218.
Syn: Achorutesarmatavar. nov.cuspidate Axelson, 1905: Thibaud et al. 2004.
Achorutes distinguendus Bagnall, 1941: Thibaud et al. 2004.
Hypogastrura (Ceratophysella) exilis Yosii, 1956: Yosii 1965.
Hypogastrura (Ceratophysella) afghanistanensis Stach, 1963: Thibaud et al. 2004.

#### Studied material.

China: Tibet: Lasa, on *Ganoderma* sp., 9 specimens on slides, leg. Weiping Xiong, 27 Mar 2015.

### Ceratophysella liguladorsi

Taxon classificationAnimaliaCollembolaHypogastruridae

(Lee, 1974)

Hypogastrura liguladorsi Lee, 1974: 95.

#### Studied material.

China: Zhejiang: Wuyi, on *Lentinus* sp., 11 specimens on slides and 80 in alcohol, leg. Xin Sun, 09 Dec 2014.

##### Key to the Chinese species of *Ceratophysella*

In 2007 Wu and Yin proposed a key to the six species of the genus *Ceratophysella* known from China. Jia et al. (2010) proposed a list of 14 Chinese species of *Ceratophysella*. The present key includes 15 species. Some of the species are not sufficiently described, but the available characters given in the descriptions are sufficient to include them in the key. The characters for *C.adexilis* have been verified on type material, and for *C.communis* on fresh material from the type locality (Tokyo).

**Table:** 

1	Abd. IV with p1 chaeta shorter than p2 chaeta (A-type)	**2**
–	Abd. IV with p1 chaeta longer than p2 chaeta (B-type)	**9**
2	Dens with 6 chaetae	**3**
–	Dens with 7chaetae	**5**
3	Unguiculus as long as 1/2–1 of internal edge of claw	**4**
–	Unguiculus very short, as long as 1/3, claw without teeth	***C.zhangi* (Zhao, 1998)** 1
4	Labial palp with 4 papillae (papilla C absent), macrochaetae rather short, Th. II with p4 = s longer thanmacrochaeta p5	***C.succinea* (Gisin, 1949)**
–	Labial palp with 5 papillae (papillae A–E present), macrochaetae long, Th. II with p4 =shorter than macrochaeta p5	***C.taiguensis* Jia, Skarżyński & Li, 2010**
5	Body chaetae smooth	**6**
–	Body chaetae serrated	**8**
6	Dens with thickened chaetae	**7**
–	Dens without thickened chaetae	***C.yinae* (Yue & Fu, 2000)**
7	Four internal chaetae on dens thickened, Ant. IV with 8 dorsal sensilla	***C.baichengensis* Wu & Yin, 2007**
–	Two internal chaetae on dens thickened, Ant. IV with 7 dorsal sensilla	***C.adexilis* Stach, 1964**
8	Abd. V tergum with chaeta a2’ present, Ant. IV with simple apical vesicle, Abd. IV tergum with p5=s equal to ½ macrochaetae	***C.denticulata* (Bagnall, 1941)**
–	Abd. V tergum without chaeta a2, Ant. IV with bi- or trilobed apical vesicle, Abd IV with chaeta p5=s equal to ¾ macrochaetae	***C.communis* (Folsom, 1898)**
9	Abd. V tergum with cuticular projection or medial spines	**10**
–	Abd. V tergum without such projection or spines	**11**
10	Abd. V tergum with medial cuticular projection, dens with 7 normal chaetae, ventral sensory file (sensory rasp) with ca. 40 peg-like sensilla	***C.liguladorsi* (Lee, 1974)**
–	Abd. V tergum with chaetae p1 modified in spines, dens with 7 chaetae among which two modified, central sensory file (sensory rasp) with ca. 25–35 peg-like sensilla	***C.duplicispinosa* (Yosii, 1954)**
11	Head with chaetae d5 and sd5 modified into spines	**12**
–	Head without chaetae modified into spines	**13**
12	Length of unguiculus as 1/3 of inner edge of claw, tibiotarsi with A1 (tenent hair) short (= ½ of inner edge of claw)	***C.xiaoi* (Tamura, 1998)** (in: Tamura and Zhao 1998)
–	Length of unguiculus as 1/2 of inner edge of claw, tibiotarsi with A1 (tenent hair) long (= length of inner edge of claw)	***C.anshanensis* (Wu & Xie, 2007)**
13	Head without a pair of cornea-like convexity	**14**
–	Head with a pair of cornea-like convexity	***C.sinensis* Stach, 1964**
14	Dens with thickened chaetae, tibiotarsi with prolonged chaeta A1 as pointed tenent hair, claws with one internal tooth and two pairs of lateral teeth	***C.skarzynskii* sp. n.**
–	Dens without thickened chaetae, tibiotarsi without prolonged chaeta A1, claws without internal and lateral teeth	***C.flectochaeta* Lin & Xia, 1983**

## Supplementary Material

XML Treatment for Ceratophysella skarzynskii

XML Treatment for Ceratophysella communis

XML Treatment for Ceratophysella denticulata

XML Treatment for Ceratophysella liguladorsi

## Footnotes

following the description in Tamura and Zhao 2008
